# A Case of Levetiracetam-Triggered Rhabdomyolysis Reversed by Drug Cessation

**DOI:** 10.7759/cureus.93033

**Published:** 2025-09-23

**Authors:** Bhagya Lakshmi Devarala, Freny Patel, Sri Vidhya Gurrala, Prashant Koirala, Prince Modi, Anand Reddy

**Affiliations:** 1 Department of Internal Medicine, Texas Tech University Health Sciences Center, Odessa, USA; 2 Internal Medicine, Pramukhswami Medical College, Karamsad, IND; 3 Internal Medicine, NRI Medical College, Mangalagiri, IND; 4 Internal Medicine, Kathmandu University School of Medical Sciences, Dhulikhel, NPL; 5 Department of Nephrology, Texas Tech University Health Sciences Center, Odessa, USA

**Keywords:** clinical case report, creatine kinase, drug-related side effects and adverse reactions, levetiracetam side effect, rhabdomyolysis

## Abstract

Levetiracetam is a commonly used anticonvulsant medication, usually a first-line drug in the treatment of generalized and partial tonic-clonic seizures. Levetiracetam is well-tolerated in most instances, with common side effects including somnolence, dizziness, and headache. Rhabdomyolysis is a rare but serious side effect that can occur. Rhabdomyolysis is caused by the breakdown of skeletal muscle fibers with creatine kinase (CK) and myoglobin release into the circulation and may be a potentially life-threatening disorder if not identified and treated early. Here, we present the case of a 34-year-old male who was admitted for two episodes of tonic-clonic seizures. Initial admission labs before the first dose of levetiracetam showed a CK level of 351 U/L. Post-admission, he was started on 1 g of intravenous levetiracetam. Subsequently, his CK levels also increased exponentially in conjunction with aberrations in his liver function tests (LFTs). Once levetiracetam was discontinued and carbamazepine (another anticonvulsant medication in a different class) was commenced, his CK and LFTs further improved, suggestive of levetiracetam-induced rhabdomyolysis. The patient’s CK level was 351 U/L at presentation and rose to over 22,000 U/L by day four after starting levetiracetam. Following a change in medication, the level decreased to 3,591 U/L by the time of discharge. This case highlights the importance of closely monitoring CK in levetiracetam-treated patients to facilitate early detection and management in the case of rhabdomyolysis. If elevated CK is detected, levetiracetam withdrawal and initiation of a different anticonvulsant should be strongly considered.

## Introduction

Levetiracetam is a broad-spectrum antiepileptic medication approved for the treatment of partial, myoclonic, and generalized tonic-clonic seizures. Its main action is through binding to synaptic vesicle protein 2A (SV2A), which, other than in the brain, is also found in muscle tissue, the parathyroid, and fibroblasts [1,2]. This binding is thought to play a role in the modulation of neurotransmitter release, N-type calcium channel blockade, and antagonism of gamma-aminobutyric and glycine current negative modulators [2]. Levetiracetam is generally well tolerated and will have only mild adverse effects such as fatigue, dizziness, somnolence, or irritability, which usually develop in the first four weeks [3,4]. On rare occasions, more serious side effects have been reported, including mood changes, psychosis, pancytopenia, and even life-threatening skin reactions such as Stevens-Johnson syndrome [5]. Rhabdomyolysis is a rare but serious complication, with only a few cases reported. It typically develops within three to five days, during which skeletal muscle fibers break down, releasing substances such as creatine kinase (CK) and myoglobin into the bloodstream. This can lead to renal damage and life-threatening complications, including arrhythmias [5-7]. One hypothesis is that levetiracetam’s activity on SV2A at motor nerve terminals of slow-twitch muscle fibers can lead to hypercholinergic activity, causing muscle stress and thus rhabdomyolysis [2].

Diagnosis is typically described by a notable elevation in CK levels, more than three to five times the upper limit of normal, and the excretion of myoglobin in urine [5-7]. Despite its therapeutic level of 12-46 pg/mL and typically low risk for treatment-emergent side effects compared with other antiepileptic drugs (AEDs), the character of the relationship between the levetiracetam blood level and rhabdomyolysis remains unclear [1]. There have been case reports of levetiracetam-induced rhabdomyolysis, with normalization of CK after drug withdrawal, suggesting a reversible association [5]. While the mechanism is not known, mitochondrial failure and calcium imbalance in muscle cells have been proposed to lead to this rare response, as with other drugs that are not related, such as statins and certain antibiotics [6].

We present a case of persistent CK elevation due to rhabdomyolysis following re-initiation of levetiracetam after several years of being medication-free, during which the patient had remained seizure-free with a history of generalized tonic-clonic seizures.

## Case presentation

**Figure 1:**
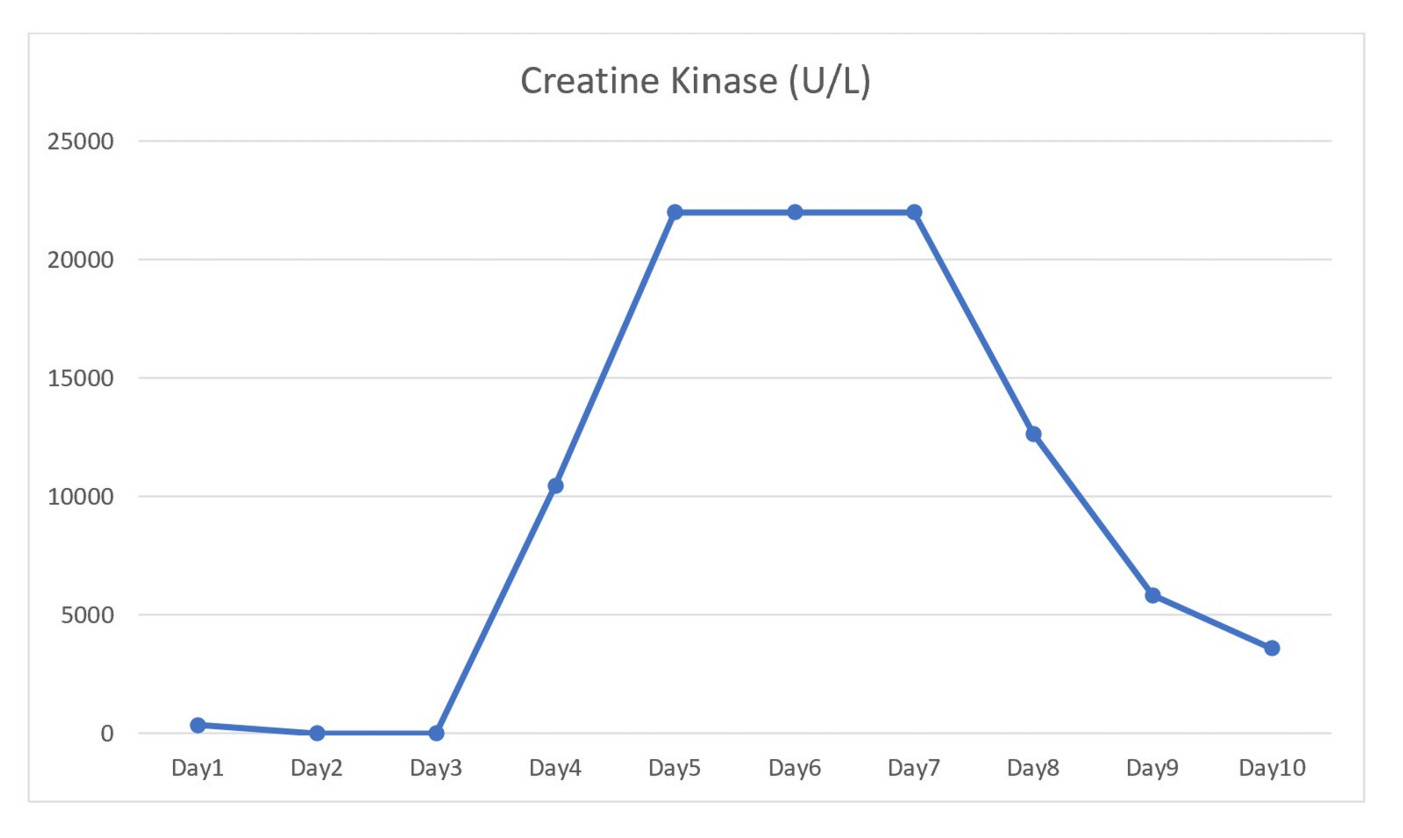
Daily creatine kinase values demonstrating rhabdomyolysis progression and resolution.

A 34-year-old male with a known history of seizures, not on any current anti-epileptic therapy, presented to the emergency department after two witnessed episodes of generalized tonic-clonic seizures, each lasting about 30 seconds. He had previously been treated with Keppra for two years, which was discontinued after he remained seizure-free for seven years. His last seizure occurred approximately nine years ago. He received Keppra 1 g intravenous (IV) loading dose followed by 500 mg IV twice daily. Due to ongoing agitation, he required emergent intubation and was admitted to the intensive care unit, later transitioning to the general medical floor after stabilization. Admission labs showed potassium of 3.4 mmol/L, blood urea nitrogen (BUN) of 10 mg/dL, creatinine of 1.2 mg/dL, ammonia of 139 µmol/L, CK of 351 U/L, aspartate aminotransferase (AST) of 27 U/L, alanine aminotransferase (ALT) of 25 U/L, alkaline phosphatase (ALP) of 60 U/L, and bilirubin of 0.2 mg/dL, with all other labs within normal limits (Table 1). Despite IV hydration, his liver enzymes and CK levels rose significantly over the next four days, with AST increasing to 532 U/L, ALT to 310 U/L, and CK peaking at 22,000 U/L (Figure 1). By day four, IV hydration increased urine output from 300-500 cc/day to 700-1,000 cc/day, accompanied by a decline in creatinine from 5.4 to 3.3 mg/dL. Electroencephalogram (EEG) showed no further clinical or subclinical seizure activity during the hospital stay. Suspecting levetiracetam-induced rhabdomyolysis, the medication was discontinued and replaced with carbamazepine. Following this change, the patient’s lab values improved, with AST decreasing to 109 U/L, ALT to 210 U/L, and CK to 3,591 U/L.The patient remained clinically stable on carbamazepine and was discharged on this medication, with plans for outpatient neurology follow-up.

**Table 1:** Key laboratory values over the hospital course.

Laboratory test	Reference Range	Day 1	Day 2	Day 3	Day 4	Day 5	Day 6	Day 7	Day 8	Day 9	Day 10
White blood cell count (10^3^/µL)	4.8–10.8	17.1	19.5	17.2	10.8	8.1	9.2	7.6	6.8	6.7	6.5
Red blood cell count (10^6^/µL)	4.7–6.1	4.88	5.09	4.58	4.17	4.25	3.35	3.85	4.25	3.95	3.99
Hemoglobin (g/dL)	14–18	14.5	15.1	13.9	12.4	12.7	9.9	11.4	12.6	11.9	11.9
Sodium (mmol/L)	136–145	143	141	140	143	142	139	144	142	143	142
Potassium (mmol/L)	3.5–5.1	3.4	3.1	4.2	4	4.3	4.5	4.1	4.3	4.4	3.7
Chloride (mmol/L)	98–107	104	106	109	110	110	104	111	110	110	110
Bicarbonate (mmol/L)	22–29	11	18	17	18	22	18	20	23	21	24
Anion gap	5–12	28	17	14	15	10	17	13	9	12	8
Creatinine (mg/dL)	0.5–0.9	1.2	1.1	4.8	5.4	3.3	1.9	1.4	1.2	1.2	1
Blood urea nitrogen (mg/dL)	6–20	10	11	21	24	23	20	15	14	12	12
Aspartate aminotransferase (U/L)	<=35	27	31	44	151	353	532	454	348	212	109
Alanine aminotransferase (U/L)	<=35	25	26	19	48	102	204	252	310	271	210
Alkaline phosphatase (U/L)	35–104	60	61	68	71	57	64	55	60	56	48
Bilirubin (mg/dL)	0.0–1.2	0.2	0.4	0.2	0.4	0.3	0.4	0.4	0.4	0.2	0.3
Creatine kinase (U/L)	<=190	351			10,453	>22,000	>22,000	>22,000	12,643	5,838	3,591

## Discussion

First approved in November 1999 as adjunctive therapy for partial-onset seizures in patients aged 16 years and older, levetiracetam is generally well tolerated, with most adverse effects reported as mild to moderate and predominantly related to the central nervous system. Its mechanism of action is unique, involving high-affinity binding to SV2A and modulation of intracellular calcium release. Despite its favorable safety profile, rare cases of rhabdomyolysis associated with levetiracetam have been reported. The exact pathophysiological mechanism remains unclear; however, it is hypothesized that interference with SV2A at motor nerve terminals may play a role [8].

Levetiracetam-induced rhabdomyolysis appears to occur in susceptible individuals, potentially due to increased sensitivity to SV2A activity in motor neurons. When rhabdomyolysis occurs, there is a release of intracellular components such as myoglobin, CK, and potassium into the bloodstream, which may result in electrolyte disturbances and renal impairment. Regular monitoring of serum CK levels, ideally before initiating levetiracetam and periodically thereafter, is essential for detecting subclinical cases and preventing renal complications [4]. A prior study reported that rhabdomyolysis may develop within 1-15 days following the initiation of levetiracetam therapy [9].

While CK levels can rise after generalized tonic-clonic seizures, typically peaking within 24-48 hours postictally [10], our patient developed marked CK elevation and rhabdomyolysis by day four of starting levetiracetam, without other identifiable risk factors such as trauma, excessive exertion, or myotoxic drugs. During this period, acute kidney injury was improving with creatinine falling from 5.4 to 3.3 mg/dL and urine output increasing with IV hydration, making seizure-related or renal causes less likely. The concurrent rise in AST and ALT, normal EEG, and subsequent CK normalization after discontinuing levetiracetam strongly suggest a drug-induced etiology [8]. This clinical course, consistent with prior case reports, supports levetiracetam-induced rhabdomyolysis as the most plausible explanation, prompting the switch to an alternative antiepileptic drug.

Most reported cases of levetiracetam-induced rhabdomyolysis have occurred within 12-36 hours of drug initiation, with peak CK levels observed three to five days later. Symptoms commonly include myalgias and dark-colored urine. In our case, CK levels peaked on day four of treatment; however, the patient remained asymptomatic and did not exhibit classic signs of rhabdomyolysis such as myalgias on dark colored urine [11]. Fortunately, most patients recover fully with prompt recognition and withdrawal of the offending agent, typically without long-term sequelae.

Clinicians should maintain a high index of suspicion for rhabdomyolysis in patients receiving levetiracetam who present with unexplained myalgias or elevated CK, especially during the initial days of treatment. As evidenced in this case and previous reports, early discontinuation of the drug usually results in symptom resolution and normalization of laboratory findings [3,8].

## Conclusions

Levetiracetam is a widely used AED, but rhabdomyolysis is an uncommon side effect. Rhabdomyolysis can lead to serious complications, such as acute renal failure, high potassium levels, and metabolic acidosis. CK levels should be checked before initiating levetiracetam and monitored periodically thereafter to allow early detection of rhabdomyolysis and timely treatment. This condition can occur in both long-term users and those just starting the medication. As patients who have tolerated levetiracetam in the past are also at risk, rhabdomyolysis should be considered in differential diagnoses during clinical evaluations.
